# Notes on the Electrical Reactions in Facial Paralysis, Especially in Reference to the Prognosis in Post-Operative Cases

**Published:** 1914-03

**Authors:** John P. I. Harty

**Affiliations:** Clinical Assistant to the Ear, Nose and Throat Departments, Royal Infirmary, Bristol


### NOTES ON THE ELECTRICAL REACTIONS IN FACIAL PARALYSIS, ESPECIALLY IN REFERENCE TO THE PROGNOSIS IN POST-OPERATIVE CASES.

BY

JOHN P. I. HARTY, B.A., M.B., B.Ch., F.R.C.S.,

*Clinical Assistant to the Ear, Nose and Throat Departments, Royal Infirmary, Bristol.*

---

IN recording these investigations on the electrical reactions in facial paralysis I do not claim originality, the main facts being stated by Sherren[1] in reference to paralysis of any motor nerve; but the point seems to have been overlooked completely by aurists, no mention being made of it in the works on otology, and the reactions generally, as far as my experience goes, are not included in the customary clinical investigations of such cases. Hence their consideration may be of some interest.

To the aural surgeon the practical value of these peculiar reactions to electrical stimulation lies in the fact that, on about the tenth day following the injury or appearance of symptoms, by carefully testing the reactions, a definite prognosis as to recovery or otherwise (*i.e.* apart from operation) may be made. There are a few exceptions to this statement which will be referred to.

The conclusions arrived at from testing on the tenth day may be briefly stated thus :—

*Loss to Faradism, with reaction of degeneration.*—Recovery, apart from operation very rare.

*Loss to Faradism, with the modified reaction of incomplete division.*—Slow recovery may be foretold.

*Loss of voluntary power, with retained power of contraction to Faradism.*—Rapid recovery.

[1] *Injuries of Nerves and their Treatment*, London, 1908.

The injury to a nerve may involve complete or incomplete division, the term "division" being used in reference to the conducting portion of the nerve. Incomplete division represents those cases with interruption or impairment of conductivity which do not lead to degeneration of the whole of the peripheral end of the affected nerve, while from a complete division total degeneration results.

The sub-grouping into anatomical and physiological varieties of complete and incomplete division is incapable of verification apart from inspection of the nerve at the site of injury.

A nerve may be incompletely severed, by a sharp cutting instrument, up to a third of its sectional area being divided, without producing any change, motor or sensory, or one of a transient nature only. This fact is of importance in the operation of nerve anastomosis.

The reactions which I refer to are the following :—

In cases of complete as compared with those of incomplete division.

A.—Stimulation with the Faradic current.

*Complete division.* No response.

*Incomplete division.* No response, except in the mildest cases, which retain their excitability to this form of stimulation.

Sherren states that rarely the muscles retain the power of voluntary contraction, although contraction to Faradic stimulation is absent, seen in cases of crutch and sleep palsies.

B.—Stimulation with the constant current.

*Complete division.* Slow, sluggish contraction.

Polar reversal ACC > KCC.

The contraction is difficult to elicit and requires a stronger current than on the normal side.

*Incomplete division.*—The contraction is sharp and brisk, as compared with that seen when the reaction of degeneration is present, but in my experience slightly more sluggish than that on the normal side.

A contraction is elicited by a weaker current than on the sound side. No polar reversal is present, KCC > ACC.

In other words, in complete division the reaction of degeneration is obtained, in incomplete an easily recognised variation is observed. The interval of ten days (strictly 8—14) is allowed for the development of these reactions.

Recovery of function in a nerve which gives the reaction of degeneration apart from operative treatment is very rare, but in the so-called cases of idiopathic facial paralysis it is justifiable to wait six months. When once the muscles cease to respond to galvanic stimulation operative treatment is useless. Before this occurs, however, years may have elapsed, thus frequent and careful testing should be made before hope is abandoned. Sherren refers to a case recorded by Purves Stewart where the muscles responded to the constant current sixteen years after the division of the nerve, and to another case of his own twenty-three years after the division of the musculo-spiral nerve.

The explanation of the reactions of incomplete division depends on the alteration in conductivity of the nerve, and the increased excitability of the motor end-plates. Hence the Faradic current, which is a stimulant of nerve fibre, loses in all except the mildest cases its power of producing a contraction, while the constant current stimulating the hyper-irritable end-plates, and not as in " R. D." the muscle fibres direct, produces a contraction with a weaker current than on the normal side, and one which is sharp and brisk when compared with that of " R. D.," which travels wavelike from bundle to bundle of fibres, starting from the point of stimulation.

My investigations to verify Sherren's statements have been carried out on cases of facial paresis supervening after the radical mastoid operation, and consequently, I cannot say unfortunately, they have not been very extensive. But in my opinion sufficient evidence of accuracy has been obtained.

It is my experience that the slightest facial twitch noticed during the performance of the operation results in an initial incomplete facial paresis (as regards the full muscular distribution of the nerve), rapidly proceeding to a complete paresis (in the same sense), which, however, in the majority of cases, on testing later, is found to be due to an incomplete division of the nerve, terminating in recovery.

With regard to treatment of these cases of incomplete division, massage stands first in importance, maintaining as it does the nutrition of the unexercised muscles. As an aid to this, artificial exercise of the muscles by means of electric stimulation is an adjunct, though its use is disputed by some. It is evident that the constant current is the one to be used, as at all events in the earlier stages the Faradic is incapable of producing a contraction, and later voluntary incipient movements are preferable to artificial stimulation. The strength of current used should be the weakest capable of producing a contraction.

The exceptions to which I referred are the following :—

1. In certain cases incomplete may pass on into complete division at a later period, as a result of compression by fibrous tissue. This indicates the importance of watching the case and taking the reactions from time to time.

2. Progressive cases due to neoplastic or inflammatory causes. Here the possibility of removal in the case of the former, or subsidence through surgical procedure in the latter, will determine the prognosis.

In the case of incomplete division the first sign of recovery is a return of voluntary power, and this is followed by excitability to Faradic stimulation. The former usually precedes the latter, often by some weeks. The muscles nearest the seat of injury are the first to show signs of recovery.

At the time when response to Faradism is regained, it is my experience that on stimulating these muscles with the galvanic current a stronger current is required to elicit a contraction than on the sound side.

I have seen one case of incomplete division which retained the power of responding to Faradic stimulation; in this instance also more milliamperage was required on the affected than on the sound side. This was one of those cases which recover rapidly.

The explanation may be that complete interruption of conductivity of the nerve fibres leads to hyper-irritability of the end-plates, impairment to a hypo-irritable state.

Theoretically it might be said that in the intact nerve the fibres convey an inhibitory influence to the end-plates. The injury acts as a stimulant to this influence, but in most cases, owing to the complete interruption of conductivity, it cannot exert its power, and so hyper-irritability is the result. However, as the nerve recovers and conductivity is re-established, or where the interruption is incomplete in the first instance, this inhibitory stimulus passes down and a hypo-irritable state of the end-plates ensues.

With regard to the times of recovery in incomplete division:—

A.—In those cases which retain Faradic excitability recovery begins in two to four weeks, and proceeds rapidly. The symptoms usually appear two or three days after the operation.

B.—In those cases which show the typical reactions of incomplete division. Recovery begins in two to nine months, and proceeds slowly. It is these cases which rarely may later develop the reactions of complete division.

I now append brief notes of a few cases.

Case I.—L. S., female, age 20.

*June 18th*, 1913.—Left radical mastoid operation and bad facial twitch noticed during operation.

Practically complete facial paralysis the same evening.

*June 25th*.—Facial paralysis complete.

*June 30th*.—Electrical reactions.

| | *Galvanism.* | *Faradism.* |
|---|---|---|
| R. | KCC>ACC. | Response. |
| L. | KCC>ACC. | No response. |

A contraction was obtained with a weaker current on the affected than on the sound side. And the contraction, although not quite so brisk as on the sound side, was not slow and sluggish as that of R. D. Unfortunately the milliamperage was not noted.

*Note*.—On the following day and subsequently (July 9th) a stronger constant current was required on the affected than on the sound side before a contraction was elicited. No polar reversal was seen on these occasions.

This presumably was during the hypo-irritable recovery phase.

| | *Galvanism.* |
|---|---|
| R. | KCC > ACC (4.5 ma. : 11 ma.). |
| L. | KCC > ACC (8 ma. : 15 ma.). |

*July 10th*, 1913.—Slight voluntary movement in upper eyelid.

*Feb. 9th,* 1914.—Facial paralysis, not completely recovered, but improving steadily.

| | *Galvanism.* | *Faradism.* |
|---|---|---|
| R. | KCC > ACC (1.2 ma.: 1.4 ma.). | Response. |
| L. | KCC > ACC (2.2 ma.: 2.6 ma.). | Response, but stronger current required. |

Also a hypo-irritable recovery phase.

Case 2.—E. R., female, age 47.

*June 25th,* 1913.—Left radical mastoid operation.

*June 27th.*—Slight facial paralysis.

*July 8th.*—Facial paralysis complete (gradually increased between these dates).

*July 9th.*—Electrical reactions.

| | *Galvanism.* | *Faradism.* |
|---|---|---|
| R. | KCC > ACC (2 ma.: 2.2 ma.). | Response. |
| L. | KCC > ACC (4.5 ma.: 4.7 ma.). | Diminished response. |

This case showed decreased irritability to galvanism on the affected side, and to elicit a contraction a stronger current was required on the affected than on the sound side.

This was a hypo-irritable state due to an initial incomplete interruption of conductivity of the nerve fibres.

*July 28th.*—Facial paralysis almost gone.

Case 3.—E. H., female, age 19.

*August 13th,* 1913.—Left radical mastoid operation. Slight facial twitch during the operation; no facial paralysis noticed the same evening.

*August 14th.*—Slight facial paralysis, which was seen to be increasing at an evening examination.

*August 26th.*—Facial paralysis complete.

*September 8th.*—Electrical reactions.

| | *Galvanism.* | *Faradism.* |
|---|---|---|
| R. | KCC > ACC (6.5 ma. : 7.5 ma.). | Response. |
| L. | KCC > ACC (2 ma. : 2.5 ma.). | No response. |

Contraction on injured side sharp and brisk, as compared with the sluggish response of R. D.

*February 9th,* 1914.—Facial paralysis practically undetectable.

| | *Galvanism.* | *Faradism.* |
|---|---|---|
| R. | KCC > ACC (1 ma. : 2 ma.). | Response. |
| L. | KCC > ACC (2.2 ma. : 3.5 ma.). | Response, but requires a stronger current. |

In the testing of February 9th there was decreased irritability to galvanism on the affected side ; a stronger current was required on the affected than on the normal side to produce a contraction.

This was also a hypo-irritable recovery phase.

Case 4.—H. R., male, aged 31.

*December 23rd,* 1913.—Right radical mastoid operation, no facial paresis noticed in the evening.

*December 24th.*—Paresis of upper and lower parts of right side of face.

*January 5th,* 1914.—No reaction to faradism.

*February 3rd,* 1914.—Electric reactions.

| | *Galvanism.* | *Faradism.* |
|---|---|---|
| R. | KCC > ACC (0.2 ma. : 0.4 ma.). | No response. |
| L. | KCC > ACC (2 ma. : 2.75 ma.). | Response. |

Contraction slower than on the normal side, but not actually sluggish.

I have to thank Dr. Watson-Williams for his kind permission in allowing me to publish the notes on these cases from his clinic at the Royal Infirmary, Bristol.

